# Epigenetic Regulation of Histone H3 Serine 10 Phosphorylation Status by HCF-1 Proteins in *C. elegans* and Mammalian Cells

**DOI:** 10.1371/journal.pone.0001213

**Published:** 2007-11-28

**Authors:** Soyoung Lee, Virginie Horn, Eric Julien, Yi Liu, Joanna Wysocka, Bruce Bowerman, Michael O. Hengartner, Winship Herr

**Affiliations:** 1 Center for Integrative Genomics, University of Lausanne, Génopode, Lausanne, Switzerland; 2 Cold Spring Harbor Laboratory, Cold Spring Harbor, New York, United States of America; 3 Program in Molecular and Cellular Biology, Stony Brook University, Stony Brook, New York, United States of America; 4 Institute of Molecular Biology, University of Oregon, Eugene, Oregon, United States of America; Netherlands Cancer Institute, Netherlands

## Abstract

**Background:**

The human herpes simplex virus (HSV) host cell factor HCF-1 is a transcriptional coregulator that associates with both histone methyl- and acetyltransferases, and a histone deacetylase and regulates cell proliferation and division. In HSV-infected cells, HCF-1 associates with the viral protein VP16 to promote formation of a multiprotein–DNA transcriptional activator complex. The ability of HCF proteins to stabilize this VP16-induced complex has been conserved in diverse animal species including *Drosophila melanogaster* and *Caenorhabditis elegans* suggesting that VP16 targets a conserved cellular function of HCF-1.

**Methodology/Principal Findings:**

To investigate the role of HCF proteins in animal development, we have characterized the effects of loss of the HCF-1 homolog in *C. elegans*, called Ce HCF-1. Two large *hcf-1* deletion mutants (*pk924* and *ok559*) are viable but display reduced fertility. Loss of Ce HCF-1 protein at reduced temperatures (e.g., 12°C), however, leads to a high incidence of embryonic lethality and early embryonic mitotic and cytokinetic defects reminiscent of mammalian cell-division defects upon loss of HCF-1 function. Even when viable, however, at normal temperature, mutant embryos display reduced levels of phospho-histone H3 serine 10 (H3S10P), a modification implicated in both transcriptional and mitotic regulation. Mammalian cells with defective HCF-1 also display defects in mitotic H3S10P status.

**Conclusions/Significance:**

These results suggest that HCF-1 proteins possess conserved roles in the regulation of cell division and mitotic histone phosphorylation.

## Introduction

Regulation of cell proliferation is central to animal development and involves the orchestration of molecular events that coordinate the cell cycle. A regulator of multiple steps of human-cell proliferation is the abundant transcriptional coregulator protein HCF-1, so called because it was first discovered as a host-cell factor utilized by herpes simplex virus (HSV) to initiate viral transcription (reviewed in [1]). During HSV infection, HCF-1 promotes formation of a multiprotein-DNA transcriptional complex with the viral protein VP16 and a second cellular protein called Oct-1 to activate HSV immediate-early gene transcription upon lytic infection.

Viruses often target key regulators of cellular function to promote infection (e.g., pRb and p53 by DNA tumor viruses), hence our interest in elucidating the natural functions of HCF-1. Genetic studies with a temperature-sensitive HCF-1 mammalian tissue-culture cell line called tsBN67 [2], [3] as well as siRNA-induced loss-of-function studies [4] have revealed that HCF-1 plays multiple roles in cell-cycle progression—specifically during the G1 and M phases.

Mature human HCF-1 is a heterodimeric complex of stably associated amino- (HCF-1_N_) and carboxy- (HCF-1_C_) terminal subunits that are generated by proteolytic processing of a 2035-amino-acid precursor [5]–[7]. HCF-1 functions in G1- and M-phase progression are largely segregated in the two subunits: In the absence of HCF-1_N_-subunit function, mammalian cells enter a stable G1-phase arrest [2], [4] and in the absence of the HCF-1_C_ subunit cells proliferate but display multiple M-phase defects including defective regulation of histone H4 lysine 20 (H4K20) methylation, chromosome segregation, and cytokinesis, the latter resulting in multinucleated cells [4], [8].

Biochemical studies of human HCF-1 have shown that HCF-1 associates with multiple histone modifying activities, including the mixed-lineage leukemia (MLL) family of histone H3 lysine 4 (H3K4) methyltransferases, MOF1 histone acetyltransferase (HAT), and Sin3 histone deacetylase (HDAC) [9]–[12], suggesting a broad role in chromatin modification. Nevertheless, except for a role of HCF-1 with the MLL family of H3K4 methyltransferases in G1-S phase progression [13], little is known of HCF-1 in chromatin modification in vivo.

Certain molecular aspects of HCF-protein function have been conserved during animal evolution. For example, the fly *Drosophila melanogaster* [14], [15] and the worm *Caenorhabditis elegans* [16] each contain an HCF-1 homolog that can stabilize the VP16-induced complex [17], [18] and the fly [15], but not the worm [19], HCF homolog retains the heterodimeric structure of proteolytically processed polypeptides. Native *Drosophila* HCF protein has been shown to associate with a Gcn5-related HAT complex [20] and overexpression studies suggest that *Drosophila* HCF can associate with H3K4 methyltransferase complexes and Sin3 histone deacetylase [21]. The roles of these complexes in *Drosophila* HCF function, however, are not known. Thus, although the cellular and biochemical properties of HCF-1 have been extensively analyzed, to date except for recent genome-wide RNAi screens, which have implicated the *hcf-1* gene in vulval development [22] and RNAi [23], the role(s) of HCF-1-related proteins in the context of an organism is poorly understood.

For a better understanding, we have undertaken a genetic analysis in *C. elegans* to study HCF-1-protein function in animal development. The *C. elegans* HCF-1-related protein is a 782 amino acid protein encoded by the *hcf-1* gene [16] and referred to here as Ce HCF-1. Ce HCF-1 is not proteolytically processed [19] but shares sequence similarity with the amino- and carboxy-terminal regions of human HCF-1 and to a lesser extent with the relatively uncharacterized human HCF-1-related protein HCF-2 [24], [25]. Here, we describe the roles of the Ce HCF-1 protein in *C. elegans* development as revealed by the analysis of two *hcf-1* deletion mutants and find that aspects of HCF-1 function in cell division and histone modification, specifically histone H3 serine 10 (H3S10) phosphorylation, are conserved.

## Methods

### *C. elegans* strains and culture

Standard culture conditions and methods were used for all *C. elegans* strains [26] unless specified. The N2 Bristol strain was used as the wild-type strain, and all strains used in this work except the *pk924* deletion mutant were provided by the *Caenorhabditis* Genetics Center (CGC, St. Paul, MN).

### Deletion mutant library screening and diagnostic PCR

For the *pk924* deletion mutant isolation, the *C. elegans* deletion mutant library and screening methods are described in [27], [28]. The library was provided by and the screening was performed while hosted by the Plasterk laboratory. The screening for the *pk924* deletion mutant was performed with two pairs of nested PCR primers specific for *C. elegans hcf-1* sequences about 3 Kb apart: LCE1 (GGACGAAGATGTCGGTTTAGAGG) and RCE1 (GTAGAACCTGGCAGTCAGCTTGC) for the first round PCR, and LCE2 (GTCGTTCGATGGCGTATTGTAC) and RCE2 (ATTCGAATCGATGATGGAGCAC) for the second round PCR. The *pk924* deletion was outcrossed with N2 worms six times. The resulting genotypes were monitored by single-worm PCR with one common 5′ primer (0.8 µM LCE1), and two 3′ primers: one *pk924* deletion-specific primer (0.4 µM RCE2) and one wild-type-specific primer (0.4 µM Ce3HCF; GATCATTCGATAAACCACCA) with cycling conditions of 92°C for 30 sec, 55°C for 30 sec, and 72°C for 30 sec for 30 cycles. The resulting wild-type 650 bp and *pk924* 817 bp PCR products were resolved by agarose-gel electrophoresis. The *ok559 hcf-1* allele was obtained from the *Caenorhabditis* Genetics Center (Accession number C46A5) and is in the RB777 strain (International *C. elegans* Gene Knockout Consortium). Single-worm *ok559* PCR genotyping was as for pk924 except that the ok559 specific PCR product is 1149 bp long.

### Immunoblot analysis

Ce HCF-1 protein immunoblots were performed with extracts from L1 larvae of N2, *pk924*, and *ok559* worms grown at 20°C as follows. Worms were collected and washed in cold 0.1 M NaCl. The worm proteins were extracted in lysis buffer (10 mM HEPES, pH 7.6 10 mM KCl, 5 mM MgCl_2_, 0.1 mM EDTA, 0.5 mM EGTA and protease inhibitors) by sonication using a Bioruptor™ 200 (Diagenode) for 5 cycles of 60 sec ON and 30 sec OFF. The lysates were cleared by microcentrifugation for 15 min at 4°C and 14,000 g. The samples were then boiled in Laemmli buffer and fractionated by 12% SDS-PAGE. The different Ce HCF-1 polypeptides were detected with the amino-terminal αCeHCF_N16_ polyclonal antibody [16] and an Alexa Fluor® 680 goat anti-rabbit IgG (H+L) secondary antibody. The membrane was scanned on the Odyssey Infrared Imaging System (LI-COR Biosciences) at 700 nm at an intensity of 1.5.

### Electrophoretic mobility retardation assay

Electrophoretic mobility retardation assays [5] were performed using total worm extracts made by French press as previously described [19] except that 2 µg of wild-type or *hcf-1 pk924* deletion mutant extract was used.

### Brood size and hatching-rate determination

The total number of embryos from an individual hermaphrodite (brood size) and embryo hatching rate were determined as follows. First, an L4 hermaphrodite was placed on a 6 cm NGM plate seeded with a small amount of bacteria. After the worm started laying eggs, it was transferred to a fresh plate every 8 to 12 h for 15°C, 20°C, and 25°C analyses, or 24 h for 12°C analysis. The number of fertilized eggs on each plate was counted and all plates were incubated at the corresponding temperatures. Hatched larvae were counted and then removed. If any embryos remained, the plate was returned to the incubator and observed for hatched embryos everyday for the following 3 to 10 days depending on the culture temperature.

### Feeding RNAi

The *hcf-1* feeding RNAi construct pGN1-CeHCF-1_FL_ was prepared by transferring the pNCITECeHCF_FL_ XbaI-BamHI fragment containing the full-length Ce HCF-1 cDNA coding sequence [16] into pGN1. The feeding RNAi *Ceglc-7α* and *Ceglc-7β* protein phosphatase 1 (PP1) constructs were made as follows: cDNA clones for *Ceglc-7α* (yk393h9) and *Ceglc-7β* (yk150g8) were provided by Yuji Kohara (National Institute of Genetics, Japan) and cloned into the XbaI/XhoI sites of the L4440 vector [29] to generate pL4440-393 (*Ceglc-7α*) and pL4440-150 (*Ceglc-7β*).

RNAi was performed following [29]. Briefly, L3–L4 worms were placed at 20°C on plates seeded with RNAi-inducing bacteria. L3 or L4 stage F1 progeny of these worms were individually placed on NGM plates seeded with the same bacteria, either maintained at 20°C or transferred to 12°C, and the brood size and hatching rate counted as described above.

### Immunostaining of *C. elegans* embryos

Immunostaining of embryos with anti-H3S10P antibody was performed as previously described [30] with a few modifications. Embryos grown at 20°C or 12°C were obtained by cutting open gravid hermaphrodites in 1 µl of water on a slide coated with poly-L-lysine (SIGMA). Poly-L-lysine treatment was done by spreading a drop of slide-sub solution [1% poly-L-lysine-hydromide (Sigma), 0.2% gelatin, 0.02% chromium(III) potassium sulfate dodecahydrate (Aldrich Chem.)] directly on the slide. For fixation, 10 µl of 2% paraformaldehyde solution (2% paraformaldehyde, 60 mM PIPES, 25 mM HEPES [pH 6.8], 10 mM EGTA, 2 mM MgCl_2_) was dropped on the embryos and the embryos were covered with a coverslip. Excess solution was absorbed with bibulous paper at the coverslip edge until the embryos were well squashed. After 10 min fixation in a humidifying chamber at room temperature, embryos were freeze-cracked [31] and immediately submerged into −20°C dimethylformamide for 10 min. Slides were washed with Tris-Tween (100 mM Tris-HCl [pH7.5], 200 mM NaCl, 0.1% Tween-20) three times 10 min and incubated with blocking solution (3% BSA, 2 mM MgCl_2_, 0.1% Tween-20) for 30 min. After blocking, embryos were incubated at 4°C overnight with anti-H3S10P antibody (Upstate Biotech.; 1∶250 dilution Abcam ab5176 ; 1∶100), washed three times in Tris-Tween, and incubated with FITC or Alexa-Fluor 546-conjugated goat anti-rabbit IgG (Jackson Laboratory, 1∶200 dilution) for 3 hr at room temperature. DAPI was added to the final wash before mounting. Samples were mounted in Vectashield Mounting medium containing DAPI (Vector lab). Embryos were similarly stained with Y1 anti-Ce HCF-1 monclonal antibody [19] to reveal Ce HCF-1 expression.

### Immunostaining of tsBN67cells

Immunostaining of tsBN67_HR1_ cells [3], [32] was performed as previously described [33] with some modifications. Prior to mounting, all procedures were performed in the original culture dishes used for cell growth on coverslips. The coverslips were first washed once with PBS and fixed in 1 ml of freshly made paraformaldehyde fixing solution (2% paraformaldehyde [Prill form, Electron Microscopy Sciences] in PBS [pH 7.4]) for 15 min at room temperature. Then they were rinsed and washed twice in PBS for 10 min. Cells were permeabilized with 0.2% Triton X-100 in PBS (pH 7.4) containing 1% normal goat serum (called PBS-NGS) for 15 min on ice, and then washed three times in PBS-NGS for 10 min. Twenty µl of anti-H3S10P antibody diluted 1∶200 in PBS-NGS was dropped on the coverslip and a small piece of Parafilm was placed over it to prevent drying. The tissue culture dish containing the coverslip was covered, placed in a humidifying chamber, and incubated at room temperature for 1 hr. Cells were subsequently washed in PBS-NGS three times for 10 min. and incubated with goat FITC-conjugated anti-rabbit IgG secondary antibody (Jackson Laboratory) diluted 1∶200 in PBS-NGS as for the primary antibody for 1 hr. Cells were subsequently washed with PBS three times for 10 min. DAPI (1 µg/ml) was added in the final wash. Coverslips were mounted with a drop of mounting medium (90% Polyscience's glycerol, 10% PBS buffered with 0.5 M carbonate-bicarbonate buffer to pH 9.0, 0.1% p-phenylenediamine).

### *C. elegans* microscopy

For the time-lapse video analysis, gravid hermaphrodites grown at 12°C were dissected in M9 buffer pre-equilibrated to 12°C. Early embryos were collected with a mouth pipette and mounted on a 2% agarose pad with small amount of M9 buffer. A coverslip was gently placed over the agar pad, and was sealed with melted Vaseline to prevent its movement. A peltier block was used to keep the stage temperature at 12°C. DIC images were captured at 7-second intervals using a Dage MTI VE1000 digital camera under the control of Scion Image software. The data were transferred to Quicktime software to make movies (played at the speed of 8 images/second).

Confocal immunofluorescence images were acquired on a Confocal Microscope Zeiss LSM 510 Meta Scanhead using a Plan-APOCHROMAT 63x/1,40 Oil objective and the Zeiss LSM 510 software. Image acquisition parameters (e.g., Laser power, pinhole aperture, photomultipliers and offset) were established with wild-type embryos grown at 12°C and 20°C stained with the anti-H3S10P antibody ; images of mutant embryos were prepared using the same parameters. Quantification of the H3S10P defect was performed with a Leica-DM6000 fluorescence microscope by identifying the percentage of stained embryos with levels of H3S10P fluorescence staining below that displayed by wild-type embryos at 12°C and 20°C.

## Results

### *hcf-1* deletion mutant isolation

**Figure 1:**
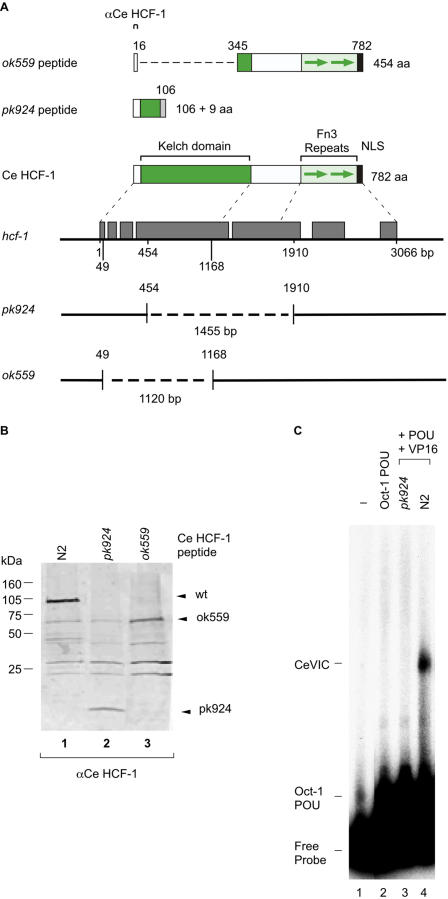
*C. elegans hcf-1* deletion mutants. (A) A schematic diagram of the *C. elegans hcf-1* gene and deletions with predicted protein structures. The *hcf-1* gene is shown in the middle with gray boxes indicating coding segments. Genomic distances from the start to end of the coding sequence along with deletion end points are indicated. The 782 amino acid Ce HCF-1 protein is shown illustrated above the *hcf-1* gene schematic showing conserved Kelch domain, fibronectin type 3 (Fn3) repeats, and nuclear localization signal [35]. The residues flanking the deletions (454 and 1910 for *pk924*, and 49 and 1168 for *ok559*) are indicated below. The *ok559* deletion is in frame whereas the *pk924* deletion creates an in frame stop codon 27 bp after the deletion junction creating the predicted polypeptides illustrated at the top. (B) Immunoblot analysis of Ce HCF-1 expression in N2 (lane 1), *pk924* (lane 2), and *ok559* (lane 3) L1-stage worms using the anti-Ce HCF-1 antisera. The lanes are from a single immunoblot in which intervening lanes have been removed. (C) The absence of HCF activity in the deletion mutant tested by electrophoretic mobility retardation assay. Electrophoretic mobility retardation analysis of VP16-induced complex formation with probe alone (lane 1) or with added Oct-1 POU domain, VP16, and *pk924* or wild-type N2 worm extracts as indicated (lanes 2–4). Mobilities of the free probe, and Oct-1 POU domain (Oct-1 POU) and VP16-induced (CeVIC) complexes are indicated.

Two *C. elegans hcf-1* deletion mutants were used in this study: *pk924* and *ok559*. *pk924* was isolated first and derives from a library of chemically mutagenized *C. elegans* [27] screened by PCR. As shown in Figure 1A, it contains a 1455 bp deletion that creates a nine-codon out-of-frame extension after the first 106 *hcf-1* codons, predicting a 115-amino-acid Ce HCF-1-related peptide. Such a Ce HCF-1 truncation protein contains only one-fourth of the conserved amino-terminal “Kelch” domain. The Kelch domain consists of six tandem Kelch repeats, each of which forms a single “blade” of a closed-circular β-propeller structure [34]—by missing more than four of these repeats, the β-propeller structure can no longer form and thus this predicted truncated Ce HCF-1 polypeptide is unlikely to retain activity.

*ok559* contains a 1120 bp long in-frame deletion that encodes a 454 amino acid protein missing nearly the entire wild-type Kelch domain (residues 17 to 344; Fig. 1A). The initial studies were performed with the *pk924* mutant and in many cases results were subsequently reinforced with the *ok559* mutant. Both homozygous mutants, while revealing defects described below, display viability at 20°C under normal growth conditions.

The absence of wild-type Ce HCF-1 protein in homozygous *pk924* and *ok559* worms was confirmed by immunoblot analysis using a polyclonal Ce HCF-1 antisera (see Fig. 1A top). This antisera recognized an HCF-1-related molecule in embryo extracts from both the wild-type and two mutant strains as shown in Figure 1B. As previously described [19], the wild-type Ce HCF-1 protein migrated at a molecular weight of about 100 kD (lane 1). In the *pk924* mutant, a small polypeptide probably corresponding to the predicted 115-amino-acid *pk924* peptide was detected (lane 2) and in the *ok559* mutant an approximately 65 kD polypeptide (comigrating with a nonspecific polypeptide) probably corresponding to the predicted 454-amino-acid *ok559* polypeptide was detected (lane 3). Thus, each predicted truncated polypeptide is accounted for in the respective mutant worm.

### Homozygous *pk924* worms lack VP16-induced complex forming activity

One of the surprising features of HCF activity is that extracts from all wild-type animal species tested have the ability to stabilize the HSV VP16-induced complex, consistent with the preservation of this aspect of HCF-protein function [17], [18]. In mammals, two proteins have been shown to possess VP16-induced complex stabilizing activity: HCF-1 and HCF-2 [24], [25]. Here, we took advantage of the *pk924* deletion to determine whether the *hcf-1* gene encodes the only HCF activity in *C. elegans* grown under normal laboratory grown conditions. Thus, we performed a VP16-induced complex formation assay with *pk924* worm extracts as shown in Figure 1C. Addition of recombinant Oct-1 POU DNA-binding domain to the HSV VP16-responsive element DNA probe results in an Oct-1 POU domain-DNA complex (compare lanes 1 and 2). Further addition of wild-type (lane 4) but not the homozygous *pk924* (lane 3) worm extract resulted in the formation of a VP16-induced complex (CeVIC). Thus, in normal laboratory grown worms, Ce HCF-1 is likely the only protein with VP16-induced complex forming activity.

### *hcf-1* mutant worms have small brood sizes and display embryonic lethality at low temperature

**Figure 2:**
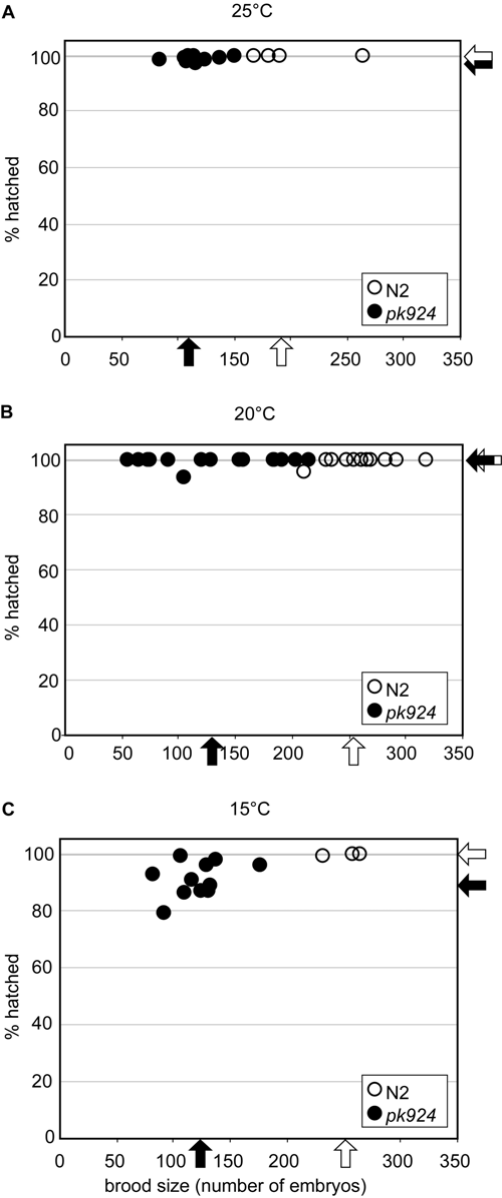
*pk924* mutant worms have small brood sizes and cold-sensitive embryonic lethal phenotypes. The results of brood size and progeny analysis at three different temperatures are shown: (A) 25°C, (B) 20°C, and (C) 15°C. The X-axis of the graphs represents the brood size, which is the number of progeny of one worm. The Y-axis represents the percentage of viable embryos in the progeny of one worm (% hatched). A filled circle represents a *pk924* worm and an open circle represents a wild-type (N2) worm. Arrows indicate the average brood size and hatching rate of either *pk924* worms (solid arrows) or wild-type worms (open arrows).

As aforementioned, the *hcf-1* deletion mutants are homozygous viable. Therefore, as a first approach to study their phenotype, we determined whether there was any change in fertility or embryonic viability with *pk924* worms at different temperatures as shown in Figure 2. We assessed fertility by measuring the brood size (i.e., the total number of fertilized eggs laid by individual hermaphrodites) and embryonic viability (i.e., the percentage of eggs that hatched).

At all three temperatures tested—15°C, 20°C and 25°C—the brood size of the *pk924* worms was about 50% that of the wild-type worms (Fig. 2A–C and Table 1), suggesting a non-temperature sensitive fertility defect upon loss of Ce HCF-1 function. Additionally, we observed weak higher incidence of males (*Him*; Table 1) and uncoordinated (*unc*; data not shown) phenotypes at all three temperatures. In contrast, the hatching rate was temperature sensitive: Whereas under our assay conditions at 20°C and 25°C the hatching rate of *pk924* worms was near 100%, at 15°C only 90% of the embryos hatched (Fig. 2C; Table 1).

**Table 1:** Progeny, viability, and incidence of males analysis at 25, 20, and 15 degrees.

Temperature	Genotype	Number of hermaphrodites	Number of progeny	Hatched embryos	Unhatched embryos	% hatched embryos	% males
25 degrees	N2	4	197±45	197±45	0±0	100±0	0.5±0.5
	*pk924*	9	112±20	111±20	1±1	99±1.0	0.8±0.8
20 degrees	N2	15	253±36	252±37	0.8±3	99.6±1.5	0.04±0.12
	*pk924*	15	124±54	124±55	0.4±1.6	99.6±1.6	0.8±1.1
15 degrees	N2	3	251±17	250±18	1±1	99.6±0.4	0±0
	*pk924*	11	121±25	110±27	11±6	90±6.0	0.8±1.3

**Figure 3:**
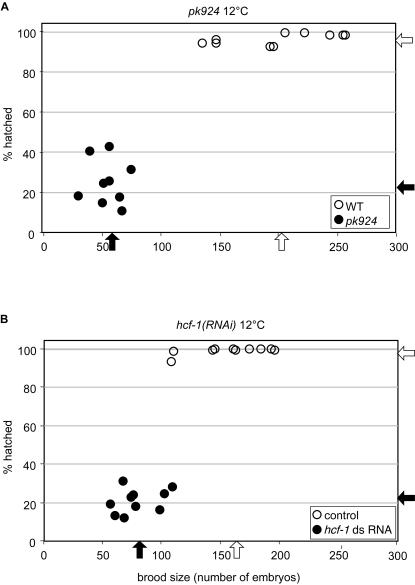
The *hcf-1* mutant phenotype is more penetrant at 12°C and is reproduced by *hcf-1* RNAi. Side-by-side comparison of the phenotypes of *pk924* worms (A) and N2 wild-type worms fed with *hcf-1* RNAi (B) at 12°C. (A) The number (brood size on the X axis) and viability (hatched % on the Y axis) of progeny were analyzed in *pk924* (solid circles) and wild-type (open circles) worms at 12°C. Arrows indicate the average brood size and hatching rate of either *pk924* worms (solid arrows) or wild-type worms (open arrows). (B) Analysis as in (A) of wild type worms fed with either *hcf-1* RNAi bacteria (solid circles and arrows) or bacteria transformed with the control empty pGN1 RNAi vector (open circles and arrows). Analyses represented in A and B were performed in parallel.

Observing some embryonic lethality at 15°C, we tested the effects of *pk924* growth at 12°C as shown in Table 2 (see also Fig. 3A for a separate experiment). At this temperature the mutant brood size was about 25% that of wild type and the mutant hatching rate decreased to about 40% (or 25% in Fig. 3A) whereas the wild-type hatching rate remained near 100%. We conclude that the homozygous *pk924* mutant has a reduced brood size across a broad spectrum of temperatures and a cold-sensitive embryonic lethal phenotype. As shown in Supporting Figure S1, the 20°C and 12°C brood size and embryonic lethality phenotypes were also observed with the *ok559* deletion mutant (See Method S1). Furthermore, the *pk924* and *ok559* mutants failed to complement each other at 12°C (Supporting Fig. S1), indicating that the observed phenotypes are indeed due to the *hcf-1* deletions.

**Table 2:** Progeny and viability analysis at 12 degrees.

Generation	Genotype	Number of hermaphrodites	Number of progeny	Hatched embryos	Unhatched embryos	% hatched embryos
First generation at 12 degrees	N2	6	123±45	122±44	1.7±2.0	98.8±1.3
	*pk924*	12	32±25	17±16	15±10	43±19.6
Third generation at 12 degrees	N2	10	253±90	25±90	3±2	98.7±1.1
	*pk924*	3+7*	99±18	33±14	66±6	33±8

* Seven hermaphrodites were sterile. Averages are for the three fertile hermaphrodites.

To determine the effects of prolonged propagation at reduced temperature, ten *pk924* worms grown for three generations at 12°C were analyzed for brood size and embryo viability. Seven of these ten worms were sterile (Table 2). The remaining three worms were fertile but had small brood sizes and hatching rates relative to wild-type worms that were similar to the first generation worms (Table 2). Thus, the third generation 12°C *pk924* worms were either sterile or displayed first generation 12°C *hcf-1* deletion mutant brood sizes and embryonic lethality, suggesting that fertility and sterility of these worms represent two epigenetic states as a result of the loss of Ce HCF-1 function.

### Feeding *hcf-1* RNAi phenocopies the *hcf-1* deletion mutants

To characterize the loss of Ce HCF-1 function phenotype further, we performed *hcf-1* feeding RNAi [29] of wild-type worms along side the pk924 mutant worms as shown in Figure 3. *hcf-1* feeding RNAi at 20°C caused reduced fertility but did not cause any embryonic lethality (data not shown). Consistent with the *hcf-1*-deletion mutant analysis (Fig. 3A), the *hcf-1(RNAi)* worms have a smaller brood size (50% of wild type) and lower hatching rate (21% vs. 99%) at 12°C (Fig. 3B). Thus, *hcf-1(RNAi)* phenocopies the *hcf-1* deletions at 12°C and 20°C, suggesting that the reduced brood size and the cold-sensitive embryonic lethality at these temperatures are both *hcf-1* null-allele phenotypes. Consistent with this hypothesis, *hcf-1* feeding RNAi of the mutant alleles did not obviously exacerbate the observed phenotype (V. Horn and W. Herr, unpublished observation).

### Ce HCF-1 immunofluorescence reveals broad embryonic Ce HCF-1 synthesis and nuclear localization

**Figure 4:**
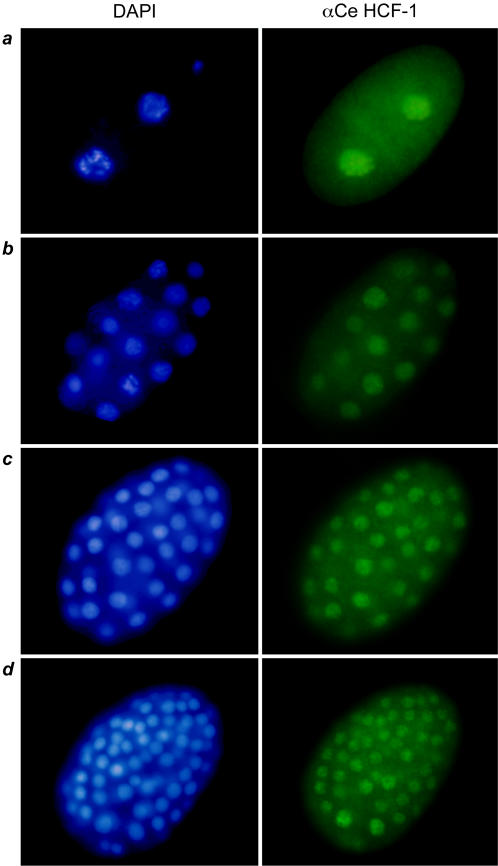
CeHCF-1 is a nuclear protein widely expressed in embryos. Immunofluorescence analysis of Ce HCF-1 expression in wild-type embryos at four progressive embryonic stages (*a*–*d*). Nuclei were visualized by DAPI staining (blue labeling, left). Ce HCF-1 (green labeling, right) was detected using the monoclonal αCe HCF-1 antibody.

The cold-sensitive embryonic lethal phenotype of the two *hcf-1* deletion-mutant and *hcf-1(RNAi)* worms suggested a role for Ce HCF-1 in embryogenesis. Therefore, we assayed the presence of Ce HCF-1 in embryos by immunostaining as shown in Figure 4. Ce HCF-1 protein was detected in all embryonic cells, mainly in the nucleus, as would be predicted by its consensus C-terminal nuclear localization signal (NLS; see Fig. 1A). The nuclear localization of native Ce HCF-1 is consistent with the nuclear localization of a recombinant Ce HCF-1–GFP protein observed in worms by Izeta et al. [35] upon heat shock-induced ectopic expression. The embryonic expression pattern described here is consistent with a role for Ce HCF-1 in early embryogenesis and the embryonic lethality observed upon loss of Ce HCF-1.

### Analysis of early-stage embryos by time-lapse video microscopy shows cell-division defects in *pk924* mutant embryos at low temperature

**Figure 5:**
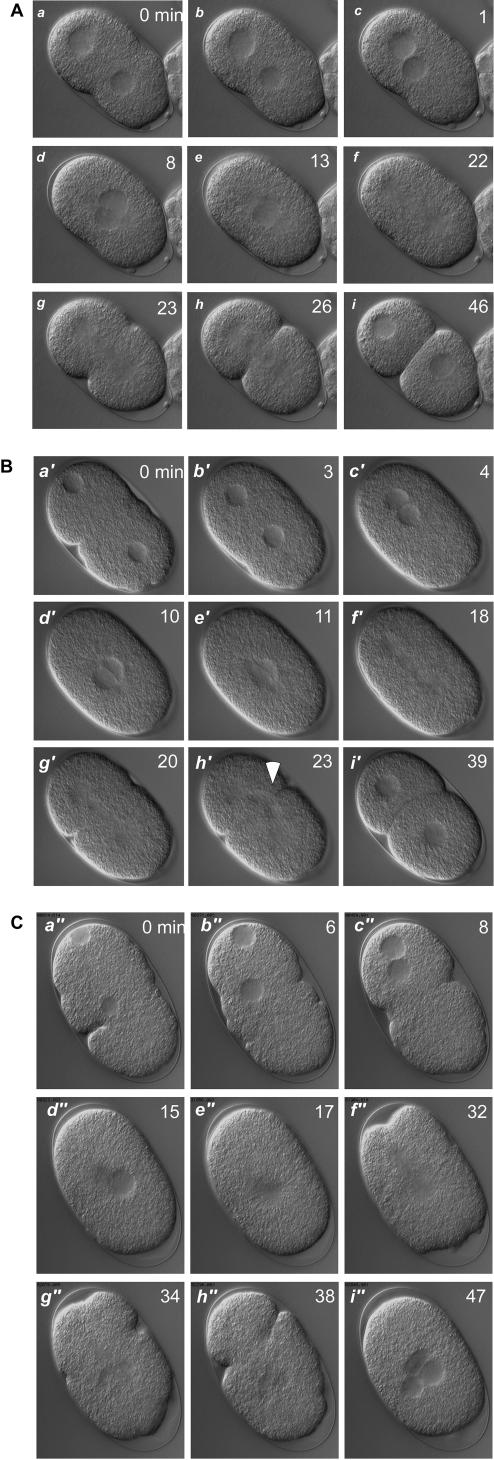
*hcf-1* mutant embryos have cell-division defects. Excerpts from embryo movies (see Supporting Information). Embryos were prepared from adult hermaphrodites grown at 12°C and kept at 12°C during the observation. The lower right corresponds to the anterior pole of each embryo. The time in minutes is indicated at the top-right corner of each panel. (A) Wild-type embryo. Under these conditions, wild-type embryos undergo slow but normal cell division and develop into normal adults. *a* to *c*, pronuclear migration; *d*, pronuclear fusion and rotation; *e*, nuclear envelope breakdown; *f*, first mitotic spindle formed along the A-P axis; *g* and *h*, first cytokinesis; *i*, two-cell stage embryo. (B) *pk924* embryo with subtle mitotic defect. Panels as in (A), note the abnormal cell division in panel *h′* (white solid arrow). (C) *pk924* embryo with severe mitotic defect. Panels as in (A) except that panel *f″* displays an inappropriate spindle, panels *g″* and *h″* display failed cytokinesis, and panel *i″* displays the multinucleated single cell embryo product.

The observation that many of the *hcf-*1 mutant embryos fail to hatch at 12°C indicated that there are defects in their embryogenesis. Microscopic examination revealed that worms carrying either mutant *hcf-1* allele when grown at 12°C frequently display cells with multiple nuclei of heterogeneous size, extra spindle poles and improper spindle alignment; the embryos often stopped development at or prior to gastrulation (data not shown). We also observed abnormally large as well as elongated eggs with weak shells. Here, we have focused on the defects in very early embryogenesis by analyzing wild-type and *pk924* mutant embryos immediately after fertilization by time-lapse DIC video microscopy with a temperature-cooled objective stage at 12°C. Selected timed frames of the movies are presented in Figure 5 and movies of the analysis are available as Supporting Information.

At 12°C, the early embryonic cell divisions of wild-type worms are slower than at 20°C but follow the normal course (Fig. 5A; Movie S1). In contrast, the two *pk924* embryos shown displayed mitotic defects ranging from mild (Fig. 5B; Movie S2) to severe (Fig. 5C; Movie S3). In Figure 5B, the chromosomes of the daughter nuclei were pushed to one side of the embryo by the first division cleavage furrow (Fig. 5B, h′, see arrowhead), which normally does not happen because the chromosomes segregate before the cleavage furrow passes for cytokinesis (compare Fig. 5A g and h with 5B g′ and h′). This phenotype suggests a cold-sensitive defect in chromosome segregation in *hcf-1* mutant worms.

The more severe defect exhibited by the embryo shown in Figure 5C begins relatively normally with pronuclear migration (a″ and b″) and fusion (c″ and d″), and nuclear envelope breakdown indicating the start of the first cell division (e″). Nevertheless, the existence of a centrosome is not clear in c″ and d″ and only a weak bipolar spindle is evident (f″). The posterior aster looks very small and the distance between the two asters does not extend the length of the embryo along the A-P axis. Although small, the rocking movement of the posterior aster is very prominent in the Movie S3, demonstrating that the microtubule spindle retains some function. There is an attempt at cytokinesis (g″ and h″) but the cleavage furrow regresses, and nuclear envelopes reappear without normal chromosome segregation, leading to the appearance of a multi-nucleated one-cell embryo (i″). This pattern of defective embryonic cell division in which there is failed cytokinesis and apparent multinucleation (see [36]) is reminiscent of the defects observed upon loss of HCF-1 function in mammalian cells [3], [8], suggesting shared involvement of HCF-1 proteins in proper cell division in mammals and worms.

### The *hcf-1* deletion mutants display changes in histone H3S10 phosphorylation status

The aforementioned results indicate that the Ce HCF-1 protein normally prevents cell-division defects during early embryogenesis at reduced temperature. Interestingly, the HCF-1_C_ subunit, which is responsible for controlling cell division in mammalian cells [4]), has been shown to associate with protein phosphatase 1 (PP1; [37]), a serine/threonine phosphatase that is involved in mitosis and inhibition of mitotic H3S10 phosphorylation. Given the defects in cell division in *hcf-1* mutant worms, we examined the phospho-H3S10 (H3S10P) status in *hcf-1* mutant worms by immunofluorescence confocal microscopy.

**Figure 6:**
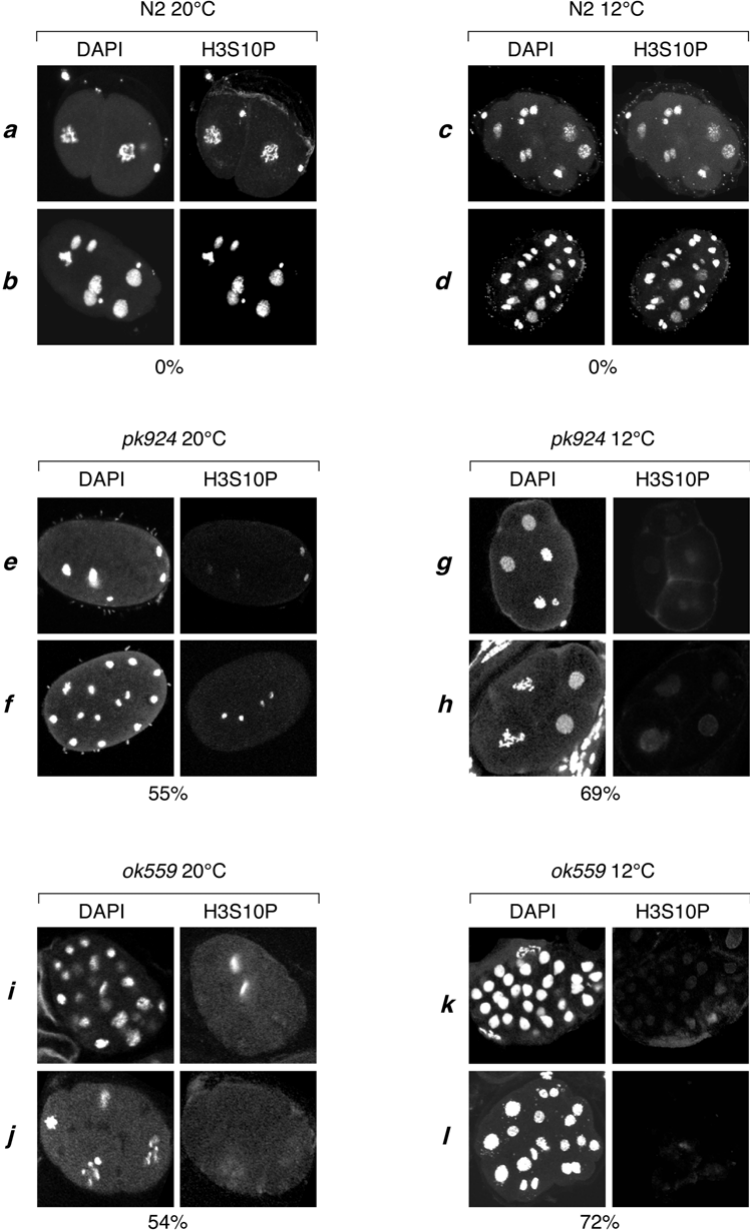
*hcf-1* mutant embryos display a reduced staining of H3S10P. The nuclei of wild-type N2, *pk924*, and *ok559* embryos collected from worms grown at either 20°C (panels a, b, e, f, i, j) or 12°C (panels c, d, g, h, k, l) were stained with DAPI (left) and anti-H3S10P antibody (right). The percentages of N2, *pk924*, and *ok559* embryos displaying reduced H3S10P staining at 12°C and 20°C are indicated below for each condition. The percentages for mutant worms were determined with the following sample sizes: N2 at 20°C, n>150; *pk924* at 20°C, n = 141; *ok559* at 20°C, n = 140; N2 at 12°C, n>150; *pk924* at 12°C, n = 150; and *ok559* at 12°C, n = 189, where n is the total number of embryos observed.

Embryos collected from worms grown at 20°C and 12°C were fixed and stained with an anti-H3S10P antibody; the DNA was stained with DAPI in parallel to identify the nuclei. As shown in Figure 6, for wild-type embryos grown at either 20°C (panel sets *a* and *b*) and 12°C (panel sets *c* and *d*), essentially all nuclei were stained with the antibody, indicating effective H3S10P modification in both interphase and mitotic nuclei. In contrast, in *hcf-1* mutant embryos grown at either 20°C (panel sets *e*, *f*, *i* and *j*) or 12°C (panel sets *g*, *h*, *k* and *l*), we observed 55 to 70% of embryos with reduced H3S10P staining, respectively. The decrease could be replicated by immunoblot analysis of bulk histones isolated under the same conditions, indicating that the decrease in H3S10P staining observed in the embryos was not due to masking of the H3S10P epitope by another molecule such as a H3S10P-binding protein (data not shown). We also did not observe embryonic H3K9 mono-, di-, or trimethylation, nor combined H3K9 trimethylation with H3S10P or H3K9 acetylation with H3S10P ‘double’ modification staining that could indicate that the decrease in H3S10P signal is due to changes in a neighboring modification that disrupts H3S10P epitope recognition (data not shown). Thus, we conclude that the reduction in H3S10P immunostaining is most likely due to a decrease in the actual levels of H3S10P modification.

As noted above, the percentage of embryos with H3S10P staining defects increased with worms grown at 12°C than at 20°C (about 70%, vs. 55%, see Fig. 6). Indeed, the penetrancy of the H3S10P modification defect at 12°C was similar to the penetrancy of the embryonic lethal phenotype at this temperature (70% vs. 65%, compare Figs. 6 and 3). These results suggest that the loss of H3S10P staining reflects changes in chromatin structure that are associated with the cold-sensitive defect in cell division.

### Mammalian cells lacking HCF-1 function also display changes in H3S10P status

Given the evident changes in *C. elegans* H3S10P detection upon loss of the Ce HCF-1 protein, we asked whether mammalian cells lacking HCF-1 function also display H3S10P staining defects. For this experiment, we used the HCF-1 temperature-sensitive hamster cell line tsBN67, which can proliferate at the permissive temperature of 33.5°C but undergoes a stable G1 cell-proliferation arrest over a period of 36 to 48 hrs at the non-permissive temperature of 40°C [2]. The arrested tsBN67 cells also display temperature-induced M-phase defects, most notably the appearance of binucleated cells, owing to disruption of HCF-1 functions in mitosis and cytokinesis [3], [4].

**Figure 7:**
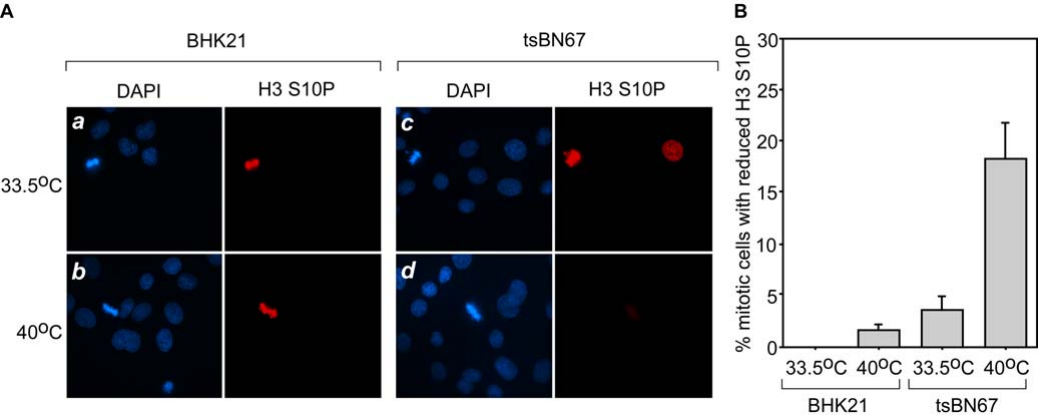
H3S10P status in mammalian cells defective for HCF-1 function. H3S10P status in BHK21 (panels *a* and *b*) and tsBN67 (panels *c* and *d*) cells visualized by immunostaining. Cells were grown at permissive (33.5°C; panels *a* and *c*) and nonpermissive (40°C; panels *b* and *d*) temperatures. Nuclei were stained with DAPI (blue labeling) and anti-H3S10P antisera (red labeling). (B) Percentage of mitotic BHK21 and tsBN67 cells displaying a reduced H3S10P staining at the permissive (33.5°C) and nonpermissive (40°C) temperatures. The results are the average of three independent samples in which for each sample 50 mitotic cells were analyzed.

We compared anti-H3S10P staining of tsBN67 cells and their wild-type parental BHK21 cells at the permissive and non-permissive temperatures as shown in Figure 7A. In these mammalian cells, the anti-H3S10P staining was much more evident in cells undergoing mitosis. We therefore analyzed mitotic cells (identified by DAPI staining) for H3S10P staining after only 24 hrs at non-permissive temperature when tsBN67 cells are still proliferating. At permissive temperature, there was robust staining of both BHK21 and tsBN67 cells (panel sets *a* and *c*); at the non-permissive temperature mitotic BHK21 cells are still stained robustly for H3S10P (panel set *b*) but many tsBN67 cells display weak staining as exemplified by the mitotic cell shown in panel set *d*. Figure 7B shows the percentage of BHK21 and tsBN67 cells displaying no or weak mitotic H3S10P staining. At permissive temperature (33.5°C), BHK21 cells did not display any mitotic H3S10P-staining defects and only a low level (about 2%) of defective H3S10P staining at non-permissive temperature (40°C). In contrast, tsBN67 cells already displayed a low level of H3S10P staining defects at permissive temperature and this level rose to nearly 20% at non-permissive temperature.

Because we only observed mitotic tsBN67 cells shortly after transfer to non-permissive temperature, our analysis may not reveal the full effects of loss of HCF-1 function on H3S10 phosphorylation. Nevertheless, these results suggest that metazoan HCF proteins share a role in chromatin modification—specifically in controlling the status of H3S10P.

### Loss of Ce HCF-1 affects H3S10P staining in the absence of PP1 function

The lack of anti-H3S10P staining in some but not all *C. elegans hcf-1* mutant embryos suggests that Ce HCF-1 is involved in but not essential for the phosphorylation process. PP1, which as aforementioned associates with mammalian HCF-1 [37], apparently inhibits H3S10P phosphorylation by (i) directly dephosphorylating H3S10P and (ii) inactivating the Aurora B H3S10 kinase, called AIR-2 in *C. elegans* [30]. It is thus possible that the effect of loss of Ce HCF-1 on H3S10P staining is owing to an enhanced activity of PP1 on dephosphorylation of the H3S10P residue or inactivation of the AIR-2 Aurora B kinase. We therefore asked whether inactivation of PP1 function would restore H3S10P staining in *pk924* mutant embryos.

**Figure 8:**
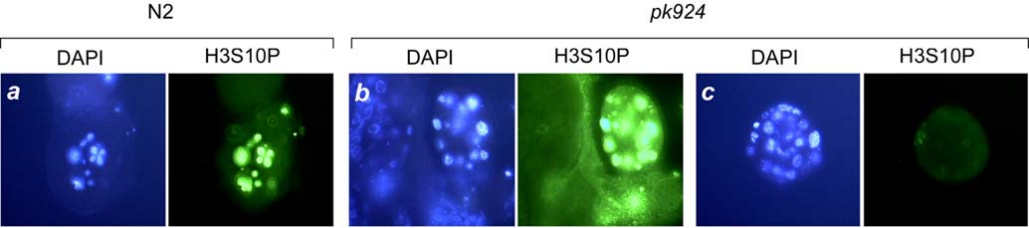
*hcf-1 pk924* embryos display H3S10P deficiency upon protein phosphatase 1 RNAi treatment. H3S10P status in N2 (panel *a*) and *pk924* (panels *b* and *c*) embryos upon RNAi of *Ceglc-7α* and *β*. Embryos were stained with DAPI (blue) and anti H3S10P antisera (green).

For this experiment, the two *C. elegans* PP1 homologs, *Ceglc-7α* and *β*, were collectively inactivated by feeding RNAi. As previously reported [30], RNAi of both *Ceglc-7α* and *Ceglc-7β* causes embryonic lethality (data not shown) and all nuclei in wild-type embryos are brightly stained with the anti-H3S10P antibody as shown in Figure 8 (panel set *a*). In *pk924* mutant embryos loss of PP1 function led to the same enhanced staining as observed with PP1 inactivation in wild-type embryos (Fig. 8, compare panel sets *a* and *b*) but did not eliminate the population of negative anti-H3S10P staining embryos (compare panel sets *b* and *c*). These results indicate that the loss of anti-H3S10P staining observed in the absence of Ce HCF-1 is not owing to an aberrant over activity of PP1. HCF proteins may have another, perhaps positive, role in maintaining H3S10P modification.

## Discussion

In this study, we have analyzed the phenotypes of two *hcf-1*-deletion alleles. The study has provided an opportunity to examine the effects of loss of HCF-protein function in a natural organismal context. Both mutants are viable and at 20°C display a relatively normal morphology, behavior, and development. Nevertheless, they also both display reduced fertility and an incompletely penetrant cold-sensitive embryonic lethality. Furthermore, they display a similarly incompletely penetrant defect in H3S10 phosphorylation that is also observed in mammalian cells upon loss of HCF-1 function. These results suggest that the Ce HCF-1 protein plays conserved regulatory but not essential roles in certain chromatin modifications and in successful development.

In contrast to the lack of a cell proliferation block that is observed in mammalian cells, the cold-sensitive cell-division defects resulting from the loss of Ce HCF-1 protein are reminiscent of loss of HCF-1 function in human cells. Nevertheless, we note that the cold-sensitivity of the *hcf-1* deletion phenotype probably reflects the temperature sensitivity of a process (e.g., mitosis) that is normally maintained by the Ce HCF-1 protein, rather than cold sensitive function of Ce HCF-1 itself. One possibility given the close percentage of penetrance of the histone modification and lethality phenotypes at 12°C is that the Ce HCF-1 protein is involved in ensuring H3S10 phosphorylation and the effects of loss of this modification are not observed except at lower temperatures.

### VP16 targets a small family of genes conserved in animals

One of the most conserved aspects of HCF proteins from different animals is their ability to interact with the HSV VP16 protein and stabilize the VP16-induced complex [17], [18]. Given that HSV is a viral pathogen, this functional conservation has most certainly not been under direct selection during evolution. Rather this functional conservation probably reflects conservation of a cellular molecular role of HCF-1 proteins that is co-opted by the virus for the purposes of regulating infection. Because deletion of the worm *hcf-1* gene leads to failure to support VP16-induced complex formation (Figure 1C), the results described here suggest that there are no other functional HCF-1 homologs in worms. Thus, the HSV VP16 protein apparently targets a critical but small gene family, HCF-1 and HCF-2 in mammals and Ce HCF-1 in worms, that has been highly conserved at the molecular level.

### Role of Ce HCF-1 in worm development

A priori, we did not expect that worms lacking Ce HCF-1 protein could sustain viability, given its important roles in regulating the cell cycle in mammalian cell culture. Other examples of such seeming discrepancies in the importance of cell-cycle regulators exist, however. For example, in mice, loss of the retinoblastoma protein pRb, an important regulator of the cell cycle, results in embryonic lethality [38]–[40]; in worms, however, the pRb homolog (encoded by the *lin-35* gene) is not required for development but, as with Ce HCF-1, its loss causes reduced fertility [41], [42]. Interestingly, HCF-1 and pRb proteins are both involved in chromatin modification and thus the reduced effects of loss of HCF-1 and pRb proteins in worms when compared to mammals may reflect differences in the role(s) of chromatin modifications in these distantly related species.

### Role of Ce HCF-1 in early embryonic cell division

Although molecular aspects of HCF-protein function are highly conserved (e.g., VP16-induced complex formation), cellular aspects seem to vary. Thus, there is no evident G1-phase arrest, a phenotype observed in mammalian cells, during worm embryonic development upon loss of *hcf-1* function. In mammalian cells, HCF-1 appears to promote G1-S phase progression by binding to E2F1 via a so called HCF-1 binding motif or HBM. The HBM is the tetrapeptide sequence ^D^/_E_HxY, where x is any amino acid, found in HCF-1 interacting proteins that is recognized by the HCF-1 Kelch domain for binding [43], [44]. Human, as well as *Drosophila*, E2F1 proteins possess the HBM and associate with human and Drosphila HCF proteins [13]. We find it interesting that the *C. elegans* E2F1 homolog, called EFL-1, does not conserve the HBM sequence. Given that loss of CeHCF-1 does not lead to a cell-cycle arrest, perhaps this E2F1-related role has not been conserved in worms.

Some relationships to E2F factors and pRb pocket proteins probably have been conserved, however, because, like the *C. elegans* pRb and E2F1 homologs, CeHCF-1 is implicated in the synthetic multivulval phenotype. Thus, *lin-35* (pRb homolog) and *efl-1* (E2F1 homolog) belong to the same class of genes called SynMuv B whose loss of function can lead to the multivulval phenotype when combined with loss of function of a gene of the SynMuv A or C group. Cui et al. [22] have shown that *hcf-1* function is important to promote the multivulval phenotype of a SynMuv AB combination suggesting that one or more relationships exist between HCF-1, pRb and E2F1 homologs from worms to humans.

In toto, these results described here suggest that HCF-1 activity has been conserved in animals for chromatin modification and cell cycle regulation.

## Supporting Information

Methods S1 Supporting Materials and Methods
(0.04 MB DOC)

Figure S1 Brood size and embryonic viability analysis at 20 and 12 degrees in pk924 and ok559 backgrounds. The progeny number (brood size) and viability (% hatched) from individual wild-type (N2), and homozygous ok559 and pk924 hermaphrodites was determined at 20 and 12 degrees. A parallel analysis of heterozygous ok559/pk924 hermaphrodites was determined at 12 degrees.
(6.54 MB TIF)

Movie S1 Initial cell divisions of wild-type embryo at 12 degrees.
(2.46 MB MOV)

Movie S2 Initial cell divisions of pk924 embryo at 12 degrees.
(2.67 MB MOV)

Movie S3 Initial cell divisions of pk924 embryo at 12 degrees displaying defective cytokinesis.
(21.96 MB MOV)
