# Supplementary material for: Epigenetic Regulation of Histone H3 Serine 10 Phosphorylation Status by HCF-1 Proteins in C. elegans and Mammalian Cells
Source: PLoS One. 2007 Nov 28;2(11):e1213. doi: 10.1371/journal.pone.0001213 (PMC2082077; doi:10.1371/journal.pone.0001213)
Supplement: Methods S1 — Supporting Materials and Methods (0.04 MB DOC) [file pone.0001213.s001.doc]

# Supporting Materials and Methods

**Method S1-Complementation test.** The heterozygous *hcf-1* deletion mutant worms (*pk924*/*ok559*) were obtained as follows: Homozygous L4 *pk924* hermaphrodites were crossed with homozygous *ok559* males and incubated at 20°C. Twenty L4 hermaphrodites of the progeny were isolated and grown at 12°C and their progeny counted and analyzed as described in Materials and Methods. The genotype of the parent was determined by single-worm PCR. The LCE1 and RCE2 primers amplify a 817 pb fragment (*pk924* allele) and 960 bp fragment (*ok559* allele) in mutant heterozogous worms.
