# Supplementary figures and images for: Epigenetic Regulation of Histone H3 Serine 10 Phosphorylation Status by HCF-1 Proteins in C. elegans and Mammalian Cells
Source: PLoS One. 2007 Nov 28;2(11):e1213. doi: 10.1371/journal.pone.0001213 (PMC2082077; doi:10.1371/journal.pone.0001213)

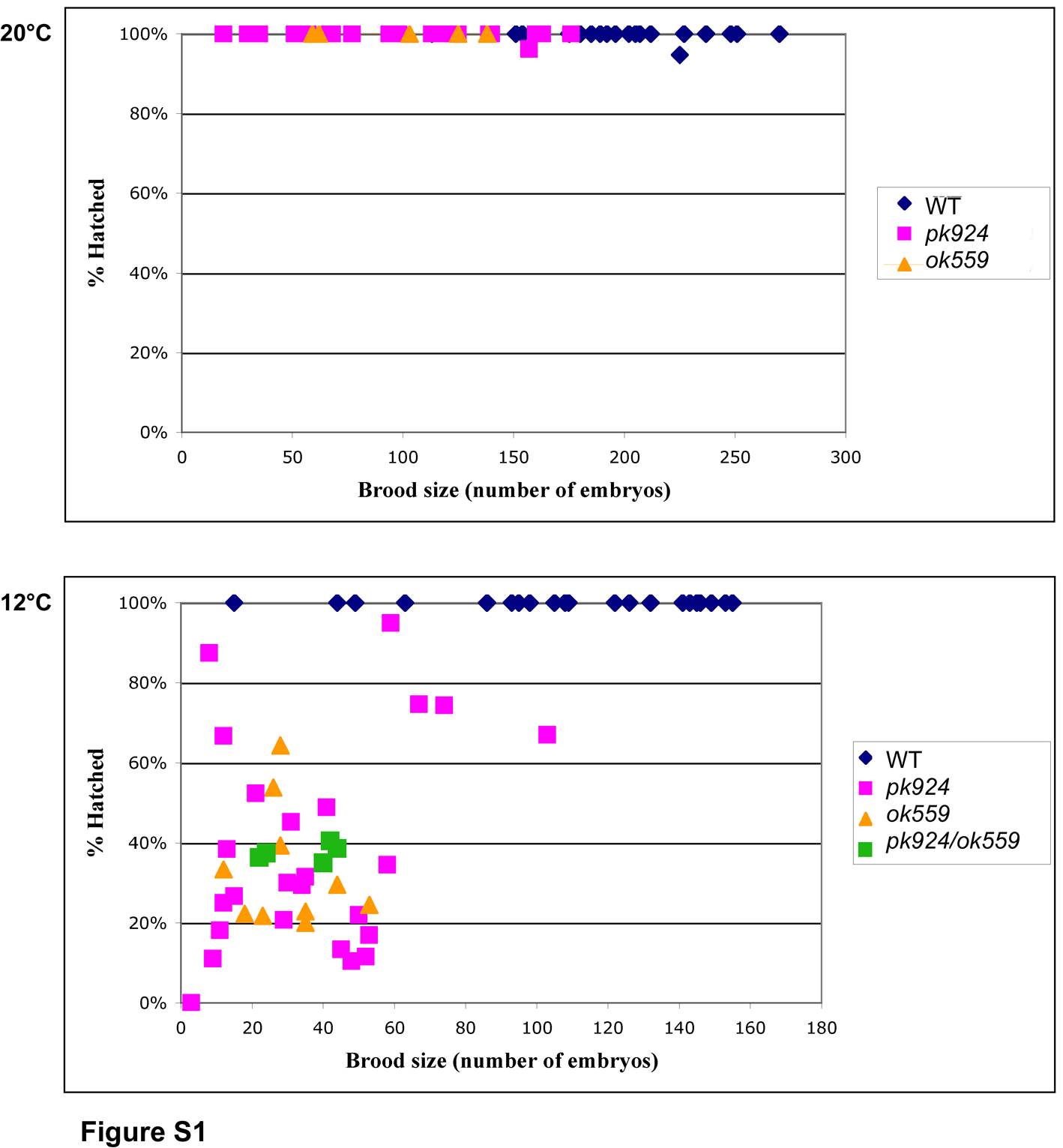

Supplement: Figure S1 — Brood size and embryonic viability analysis at 20 and 12 degrees in pk924 and ok559 backgrounds. The progeny number (brood size) and viability (% hatched) from individual wild-type (N2), and homozygous ok559 and pk924 hermaphrodites was determined at 20 and 12 degrees. A parallel analysis of heterozygous ok559/pk924 hermaphrodites was determined at 12 degrees. (6.54 MB TIF) [file pone.0001213.s002.tif]
